# Brazil's Pivotal Moment in Public Health: Establishing the Interministerial Committee (CIEDDS) for the Elimination of Tuberculosis and Socially Determined Diseases

**DOI:** 10.1590/0037-8682-0597-2023

**Published:** 2024-02-12

**Authors:** Ethel Leonor Noia Maciel, Mauro Niskier Sanchez, Alda Maria Da Cruz, Draurio Barreira Cravo, Nísia Verônica Trindade Lima

**Affiliations:** 1 Ministério da Saúde, Secretaria de Vigilância em Saúde e Ambiente, Brasília, DF, Brasil.; 2 Universidade de Brasília, Departamento de Saúde Coletiva, Brasília, DF, Brasil.; 3 Ministério da Saúde do Brasil, Gabinete da Ministra, Brasília, DF, Brasil.

The Unified Health System (*Sistema Único de Saúde*, SUS) offers universal systemic healthcare to all citizens resident in Brazil. Unfortunately, the system has also suffered years of underfunding, while Brazil is going through a very particular epidemiological transition, involving a triple challenge in the field of healthcare: at the same time as chronic non-communicable diseases, as well as accidents and violence are important public health issues, so are communicable diseases, still constituting an unacceptable burden on the health system, impacting population health.

Since President Luiz Inácio Lula da Silva took charge in January 2023, health has been treated as an absolute priority, given the country’s health status and the resurgence of diseases that were previously under control, especially those that are strongly socially determined, or transmitted by vectors, including tuberculosis (TB), leprosy, geohelminthoses, malaria, and most waterborne diseases. These ancient diseases affect a much larger number of individuals in more socially vulnerable settings, shaming us as a nation.

In response to these issues, the President signed several social and health programs, including *Mais Médicos* (More Doctors), *Brasil Sorridente* (Smiling Brazil, for dental health), *Bolsa Família* (a conditional cash transfer program), *Minha Casa Minha Vida* (My House, My Life) and *Brasil sem Fome* (Brazil without Hunger), and also signed Decree 11,494, of April 17, 2023, which established the Interministerial Committee for the Elimination of Tuberculosis and other Socially Determined Diseases (*Comitê Interministerial para a Eliminação da Tuberculose e de Outras Doenças Determinadas Socialmente*, CIEDDS)^1^. Nine ministries, coordinated by the Ministry of Health, participate on this Committee, whose purpose is to promote inter-sector actions that contribute to eliminating diseases with strong social determinants, those mainly affecting the most vulnerable populations, and those with greater difficulty accessing healthcare services.

Brazil is a signatory to the United Nations Sustainable Development Goals (SDGs), several of whose targets align with the Brazilian government’s program. The first SDG is to eliminate poverty; the second, to eradicate hunger; and the third, focusing on good health and well-being, which entails ending the epidemics of important communicable diseases. All of the goals are contained in Decree 11,494 and define the focus of the CIEDDS’s activities.

Control measures, one fundamental pillar of health surveillance, may aim to reduce incidence, case severity, mortality, or lethality, depending on the health condition in question. A disease is considered eliminated when there are no more cases, even though control efforts may need to be continued. There is, however, also the concept of elimination as a public health problem, which is when agreed targets (e.g., very low incidence) are met, while still calling for continued work towards eliminating the disease in question. A disease control program can also aim to achieve specific operational targets, which can be seen as intermediate steps towards reaching elimination, in the two senses described above. Elimination differs substantially from eradication, which entails ending not only cases of disease, but also the causes of the disease, particularly the etiological agent, thus preventing reintroduction of the disease^2^. Of the diseases covered by the government decree, leprosy and TB are ancient and have already been eliminated as public health problems in all developed countries, although they persist in the lives of the most impoverished populations in developing countries, including Brazil.

Leprosy still affects around 24,000 Brazilians every year and TB affects almost 80,000, killing around 14 Brazilians every day^3^ ^,^ ^4^. Their common characteristic is that they are associated with clusters of people and require multidrug treatment lasting more than 12 months. With respect to TB, co-infection with the human immunodeficiency virus (HIV) worsens prognoses, as it predisposes to relapses and increased drug resistance. TB/HIV co-infection is especially relevant among the incarcerated population, where TB incidence is extremely high. Among the 30 countries with the most incarcerated individuals, Brazil has the highest disease burden^5^. Both diseases, but especially leprosy, can lead to permanent sequelae. The stigma and discrimination that interfere with individuals’ sociability and even prejudice their access to the job market are also crucial aspects to consider.

Intestinal parasitic diseases remain practically invisible, although Brazil’s national survey on the prevalence of schistosomiasis and geohelminthiases^6^ points to locations with over 15% of schoolchildren infected, many of them polyparasitized. However, the real prevalence of infection by waterborne protozoa, including giardia and cryptosporidia, remains unknown, and these are among the most prevalent agents identified in global studies, such as the Malnutrition and Enteric Disease Study^7^, a multicenter study of the effects of malnutrition and intestinal infections on children in developing countries. Accordingly, the extent of the impacts on children’s physical and cognitive development is also unknown, while the prospects for elimination are poor, because low coverage by basic sanitation, especially treated water, favors frequent reinfection. The same is true of schistosomiasis, which, although concentrated in specific areas, can be eliminated only by actions that extend beyond the healthcare sector.

Intermediate elimination goals can be set for some diseases, such as Chagas disease. In 2006, Brazil received an International Certificate of Elimination of Transmission of Chagas Disease (*Trypanosoma cruzi*). Elimination of mother-to-child transmission is currently seen to be an intermediate goal achievable in the medium term. However, the increase in cases of oral transmission of *T. cruzi* observed over the past two decades poses a new challenge, with the Amazon region becoming a new frontier for Chagas disease. The biological characteristics of local vectors, as well as cultural habits, make ingestion of contaminated food a significant risk factor for trypanosomiasis in this region^8^.

Programs to combat malaria since the 1950s have managed to eliminate the disease in several parts of Brazil, restricting transmission to practically the Amazon region alone. Actions taken in the Amazon produced cycles of significant reductions in cases, which were not sustainable. This was principally because of discontinuities in funding for control measures. Recorded cases of malaria and related deaths have increased in the past two years, showing clearly that difficulties in accessing diagnosis and treatment - here, under the effects of the coronavirus disease pandemic - have had a direct impact on the burden of disease. Around 80% of malaria cases are concentrated in 33 municipalities in the Amazon region and 33% of cases occur in indigenous territories, many of which are difficult to access. This calls for a targeted approach that incorporates the cultural specificities of different ethnic groups inhabiting areas strongly affected by malaria^9^. These figures have prompted more intensive action by the Malaria Elimination Plan, whose success depends on improving access to diagnosis and treatment.

Onchocerciasis, also called “river blindness” or “miner’s disease,” is almost entirely (99%) concentrated in just over 30 countries and, in Brazil, mainly affects indigenous populations, particularly the Yanomami population in the Amazon. Likewise, trachoma, the main cause of blindness of infectious origin, occurs only in areas with higher poverty and with little basic sanitation or access to water, mainly in Brazil’s North and Northeast regions^10^.

Syphilis persists as a disease and a problem of social inequality in Brazil and is still transmitted by pregnant women to their fetuses throughout the country, as are HIV, hepatitis, and Chagas disease^11^. Access to prenatal care and rates of hospital birth are both high in Brazil, meaning that the goal of eliminating these mother-to-child diseases is feasible by 2030, and is, therefore, a CIEDDS goal.

With focus and efficiency, priority actions aimed especially at geographically concentrated diseases can eliminate these public health problems in Brazil in a short time. One good example is lymphatic filariasis (elephantiasis), which once plagued coastal populations in parts of Brazil’s Northeast and today is targeted for elimination^10^.

It is essential that elimination plans operate in a coordinated manner because several diseases may overlap in a single territory. Inter-sector actions can constitute an innovative pillar in policies to control and eliminate infectious diseases, providing they addresses common determining factors of infection and illness, including basic sanitation or ecosystem characteristics, such as the Amazon, and prioritize social determinants. With that in mind, the Ministry of Health is forming an Amazon Health Actions Working Group (Ministerial Order GM/MS No. 707, of June 7, 2023), under the coordination of the Health and Environmental Surveillance Secretariat, to oversee Ministry of Health activities as part of the Legal Amazon Health Program (*Programa de Saúde da Amazonia Legal*, PSAL).

The CIEDDS will be responsible for preparing a National Program for the Elimination of Socially Determined Diseases. This will be funded initially by the budget of participating ministries, but it will also seek additional funding from public entities or not-for-profit organizations, reinforcing the government's commitment to the health of the Brazilian population, especially the most vulnerable groups at greatest risk of falling ill from preventable diseases. This will be achieved by promoting public policies that guarantee dignified living conditions and access to the basic rights guaranteed to our citizens by the 1988 Constitution, which stipulates that health is everyone’s right and the State’s duty.
